# Left atrial volume index and aortic stiffness index in adult hemodialysed patients - link between compliance and pressure mediated by endothelium dysfunction; a cross-sectional study

**DOI:** 10.1186/1471-2261-12-100

**Published:** 2012-11-05

**Authors:** Tomasz Zapolski, Andrzej Wysokiński, Andrzej Książek, Andrzej Jaroszyński

**Affiliations:** 1 Chair and Department of Cardiology, Medical University of Lublin, Lublin, Poland; 2 Chair and Department of Nephrology, Medical University of Lublin, Lublin, Poland; 3 Chair and Department of Family Medicine, Medical University of Lublin, Lublin, Poland

**Keywords:** Left atrium volume index, Aortic stiffness, NT-pro BNP, Asymmetric dimethylarginine, End-stage renal disease, Hemodialysis

## Abstract

**Background:**

This study was performed to investigate the relationship between elastic properties of aorta and left atrium volume index (LAVI) in hemodialyzed (HD) patients.

**Methods:**

Study group was consisted of 73 patients (age 51,6 ± 7,6 years) treated by hemodialysis. In all patients standard echocardiography was performed. Aortic stiffness index (ASI) was calculated using formula: ASI = log (SBP/DBP)/[(Aomax-Aomin)/Aomin]. LAVI was calculated according to the formula: LAVI = [π/6 x (LAmax x LAshort x LAlong)]/m^2^. Additionally several indices were calculated: left ventricle mass (LVM), left ventricle mass index (LVMI), midwall fractional shortening (mFS), endsystolic stress (ESS), mFS/ESS. Additionally the laboratory parameters including lipidogram, troponin T (cTnT), NT-proBNP and asymmetric dimethylarginine (ADMA) were measured.

**Results:**

The ASI was strong and significantly correlated with left atrium volume (LAV) and LAVI (respectively: 0,601; p < 0,001 and 0,598; p < 0,001). The ASI was independently and markedly associated with ADMA, cTnT, CRP, T-chol, and LDL-chol. The LAVI was independently and significantly correlated with NT-proBNP and cTnT.

**Conclusions:**

There is correlation between ASI and ADMA, marker of endothelium dysfunction. There is also association between LAVI and NT-proBNP, signs of elevated left atrium pressure. The strong correlation between ASI and LAVI, improved by associations of specific biochemical markers with these echocardiographic indices, suggests there is the link between elastic properties of aorta and left atrium pressure in hemodialysed patients mediated by endothelial dysfunction.

## Background

Cardiovascular complications are the main cause of death in patients with end-stage renal disease (ESRD) on renal replacement therapy [1]. Most of recent advances in the explanation of cardiovascular complications related to ESRD have concentrated on atherosclerosis, and much less studies have been dedicated to evaluate the mechanism related to hemodynamic changes occurred as a consequence of renal failure and renal replacement interventions. Morphological and functional alterations in response to the overload of pressure and volume characteristic for ESRD are named as uremic cardiomyopathy.

Left atrium (LA) enlargement is a hitherto overlooked component of the complex echocardiographic alterations that are observed in ESRD. In individuals with ESRD, left atrial volume (LAV) is independently associated with angiographically significant coronary artery disease, history of stroke, carotid intima-media thickness and signs of inflammation such as high concentration of hs-CRP [2]. Left atrium volume is adjusted to body surface area and expressed as left atrium volume index (LAVI). It has been postulated to use in ESRD LAVI as a potent biomarker for risk stratification and risk monitoring in patients with ESRD [3].

Because the LA is exposed to left ventricle filling pressures through the open mitral orifice during diastole, its size is influenced by the same factors that determine diastolic filling pressure. The diastolic dysfunction of the left ventricle is a common finding among patients with ESRD. One of the most important factors influencing left ventricle function independent of blood pressure is aortic stiffness [4].

Atherosclerosis was regarded as a combination of two major separate diseases: atherosis and sclerosis. Sclerosis component depends on deterioration of aortic elastic properties and is called aortic stiffness. The stiffness of the aorta influences aortic conduit function causes pressure elevation and abnormal pressure pattern that increases the afterload of the left ventricle. In that way may induce left ventricle hypertrophy and alter on left ventricle diastolic and systolic function. It is well known that aortic stiffness is an independent predictor of all-cause and cardiovascular mortality in different group of patients [5] including patients who require renal replacement therapy [6].

Endothelial dysfunction is a crucial precursor of the development of cardiovascular disease. The endothelium maintains the balance between vasoconstriction and vasodilatation. The role of the endothelium in controlling the vascular tone, especially vasodilatation, has been shown via the endothelial-derived nitric oxide (NO) [7]. Decreased NO production has also been linked to progression of renal dysfunction [8].

### Aim

This study was performed to investigate the relationship between elastic properties of aorta and LAVI in hemodialised (HD) patients. Moreover the relationship between these functional and morphological parameters and selected biochemical markers was assessed.

## Methods

### Clinical characterization of studied groups

Study group was consisted of 73 patients (35 women and 38 men in age of 51,6 ± 7,6 years) treated by hemodialysis for mean time of 78,71(±41,04) months. Hemodialysis were performed three times a week using devices as: Fresenius 4008B/S (Fresenius medical care, Bad Homburg, Germany) and Gambro AK95S (Gambro, Lund, Sweden). Bicarbonate dialysate containing (in millimoles per litre) 32 bicarbonate, 136–138 sodium, 2.5–4.0 potassium, 1.0 magnesium and 1.25 or 1.5 calcium was used in all HD patients. The mean time of hemodialysis session was 4.09 ± 0.11 h. The diffusive technique was applied in all cases**.** During hemodialysis, no medication was applied except heparin. All patients were hemodialysed for >6 months and were clinically stable. Out of 73 HD patients, 46 (60,2%) were taking angiotensin-converting enzyme inhibitors/angiotensin receptor blockers, 41 (56,2%) beta-blockers, 44 (60,3%) calcium blockers and 24 (32,9%) statins.

The causes of ESRD in this group were as follow: glomerulonephritis – 32(43,8%), diabetes mellitus – 11(15,1%), chronic tubulo-interstitial nephritis – 3(4,1%), obstructive nephropathy – 5(6,8%), hypertonic nephropathy – 3(4,1%), polycystic kidney disease – 4(5,5%) and unknown/uncertain – 15(20,5%).

### Control group

The control group comprised of 57 volunteers (27 women and 30 men) in age of 51,9 ± 7,1 years of comparable to study group clinical characterization, except that they didn’t suffer from ESRD. They have had no abnormalities detected by physical examination, ECG, chest X-ray and laboratory analysis.

### Echocardiography

In all patient and control subjects standard transthoracic echocardiographic examination was performed using 2,5-3,5 MHz transducer (HP Sonos 7500, Hewlett Packard, Bloomfield, CT, USA) by the cardiologist, who was blinded to the clinical data of the study subjects. All echocardiographic measurements were done according to the guidelines of the American Society of Echocardiography [9]. To exclude the influence of extracellular volume condition on systemic hemodynamic, echocardiographic examination was performed during first 60 minutes after a single hemodialysis session.

The following a two-dimensionally guided M-mode echocardiographic parameters were recorded: interventricular endsystolic septum diameter (IVSSd [cm]), interventricular septum enddiastolic diameter (IVSDd [cm]), posterior wall systolic diameter (PWSd [cm]), posterior wall diastolic diameter (PWDd [cm]), left ventricle enddiastolic diameter (LVEDd [cm]), left ventricle endsystolic diameter (LVESd [cm]), left atrium maximal diameter (LAmax [cm]), aortic maximal diameter (Aomax [cm]), aortic minimal diameter (Aomin [cm]). The dimensions of the aorta were recorded in M-mode 3 cm above the aortic valve from a parasternal long axis view. The Aomax (systolic diameter) was measured at the time of aortic valve maximal opening. The Aomin (diastolic diameter) was recorded at the peak of the QRS complex. Inner aortic diameters were measured with a caliper in systole and diastole as the distance between the trailing edge of the anterior aortic wall and the leading edge of the posterior aortic wall [10]. In 4-CH presentation were additionally recorded: left atrium short diameter (LAshort [cm]), left atrium longitudinal diameter (LAlong [cm]), left atrium surface (LAS [cm^2^) and left atrium circumference (LAC[cm]). Consequently several standard indices were calculated according to American Society of Echocardiography recommendations [11] such as: left ventricle stroke volume (SV[ml]), stroke index (SI [n]), cardiac output (CO [l/min]), cardiac index (CI [l/min/m^2^), ejection fraction (EF [%]), fractional shortening of left ventricle (FS [%]). Using previously measured parameters the most important for present study indices were calculated according to formulas:

● Left ventricle mass (LVM [g]): LVM = 1,04x[(IVSDd + PWDd + LVEDd)^3^]-13,6 g

● Left ventricle mass index (LVMI [g/m^2^]): LVMI = LVM/BSA

**Figure 1:**
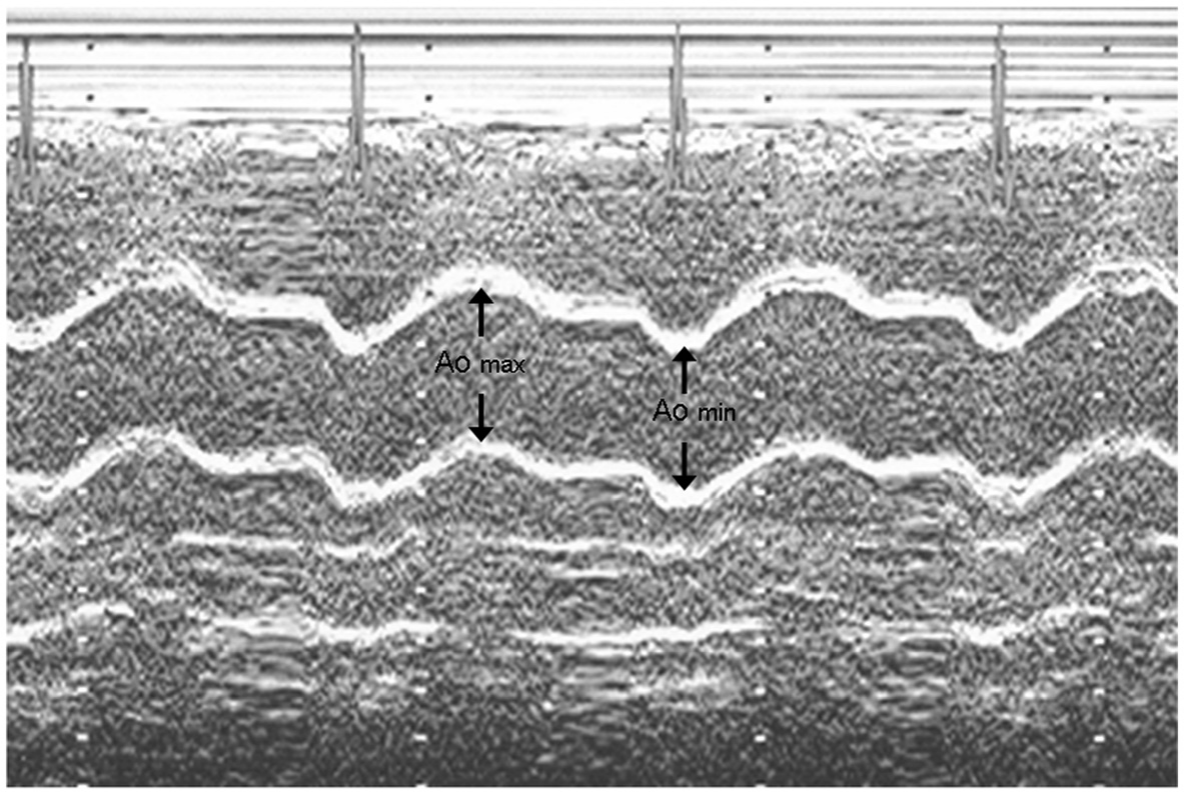
The measurement of diameters needed for ASI calculation.

● Endsystolic stress (ESS [10^3^dyn/cm^2^]): ESS = 0,334xSBPxLVESd/PWSdx(1 + PWSd/LVESd),

● Midwall fractional shortening (mFS [n]): mFS=[(LVEDd + PWSd/2 + IVSSd/2)–(LVESd + Hs/2)/(LVEDd + PWSD/2 + IVSSd/2)]x100; where Hs = IVSSd + PWSd,

● Ratio mFS/ESS [n],

● Left atrium volume (LAV[ml]): LAV = LAVI= π/6x(LAmaxxLAshortxLAlong) [12] (Figure 1),

● Left atrium volume index (LAVI [ml/m^2^]): LAVI = LAV/m^2^

**Figure 2:**
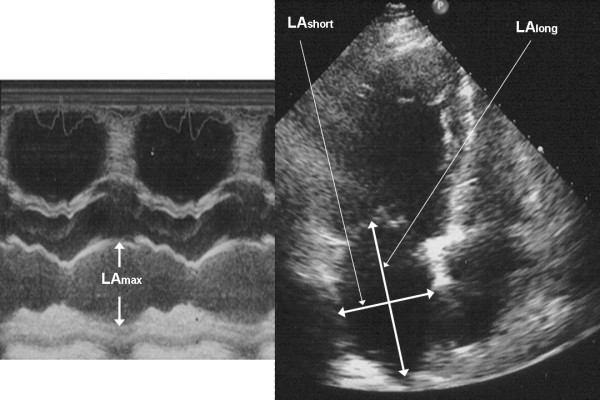
The measurement of diameters needed for LAVI calculation.

● Aortic stiffness index (ASI [n]): ASI = log [(SBP/DBP)/(Aomax-Aomin)]/Aomin [10] (Figure 2).

Left ventricle hypertrophy was defined by a LVMI of > 134 g/m^2^ in men or > 110 g/m^2^ in women.

Moreover pulsed Doppler derived parameters were measured such as: maximal velocity of early diastolic transmitral flow (E [cm/s]), maximal velocity of late diastolic transmitral flow (A [cm/s]), isovolumetric relaxation time (IVRT [ms]), maximal systolic velocity in pulmonary veins (S [cm/s]), maximal diastolic velocity in pulmonary veins (D [cm/s]). Consequently ratios of E/A and S/D were calculated. Diastolic dysfunction was recognized accordingly to recommendation of Canadian Cardiovascular Society [13]. Patients with restrictive spectrum were not included to this study.

The most important for this study echocardiographic indices were: LAVI and ASI. Patient from HD group were divided and compared using mean values of mentioned indices as cut-off values.

### Anthropometry, pressure and laboratory measurements

Body surface area was calculated according to formula: BSA (m^2^) = 0.0235 x height(cm)^0.42246^ x weight(kg)^0.51456^[14].

The systolic (SBP) and diastolic (DBP) pressures were obtained using electronic sphygmomanometer (Omron 705 CP, Omron, Kyoto, Japan). The brachial artery blood pressure measurement was made with the subjects remaining in the left lateral decubitus position in darkened, quiet room. While obtaining ascending aorta diameters by echocardiography, blood pressure in the right upper arm was simultaneously measured. Mean blood pressure (MBP) was calculated by the formula: MBP = 1/3 SBP + 2/3 DBP. Moreover the mean heart rate calculated from the consecutive 10 beats was recorded.

The following standard parameters were measured by automated analyzer (ADVIA Centaur analyzer, Bayer Health-Care Diagnostics, Tarrytown, NY, USA): hemoglobin [g/dl], sodium [mmol/l], potassium [mmol/l], calcium [mmol/l], phosphorus [mmol/l], Ca x P score [mg^2^/dl^2^], creatinine [μmol/l], glomerular filtration rate (GFR) [ml/min/1,73 m^2^], urea [mg/l], uric acid [mg/dl], protein [g/l], albumin [g/l], high sensivity C-reactive protein (hs-CRP) [mg/ml], fibrinogen [g/l], total cholesterol (T-chol) [mg/dl], HDL-cholesterol (HDL-chol) [mg/dl], triglycerides (TGC) [mg/dl]. LDL-cholesterol (LDL-chol) [mg/dl] was calculated using the Friedewald equation: LDL-chol = T-chol – HDL-chol – (TGC/5). GFR was calculated from the modification of diet in renal disease (MDRD) formula: GFR = 186xserum creatinine^-1.154^ x age^-0.203^ x F, where F = 1 in men and F = 0,742 in women. Cardiac troponin T (cTnT) [(μg/l] in plasma was measured by the electrochemiluminescence immunoassay (Elecsys 2010 analyser, Roche Diagnostics Gmb, Mannheim, Germany) with the detection limit of 0,01 μg/l. NT-proBNP [fmol/ml] in plasma was measured by the enzyme-linked immunosorbent assay - ELISA method (Biomedica, Bratislava, Slovakia) with the detection range between 0–640 fmol/ml. Asymmetric dimethylarginine (ADMA) [μmol/l] was measured by immunoenzymatic method – EIA (ALPCO Diagnostics, Windham, New Hampshire, USA) with the detection range between 0,05-3,0 μmol/l and reference range for healthy population of 0,4-0,75 μmol/l).

The study protocol was approved by a university ethics review board. The investigation conforms with the principles outlined in the Declaration of Helsinki.

#### Reproducibility

Intraobserver variability for ASI and LAVI measurements were assessed. For intraobserver variability, a sample of 10 ASI and LAVI measurements was randomly selected and examined by the same observer in two different days after sessions of hemodialysis. Intraclass correlation coefficients for the same observer were calculated [15].

#### Statistical analysis

Statistical analysis was carried out on an IBM PC using of a standard statistical package (SPSS for Windows Version 12.0; SPSS INC, Chicago, Illinois). Data were expressed as mean ± SD (parametrically distributed continuous variables) and percentage (categorical variables). The statistical significance of the differences between HD patients and control group means were compared by unpaired Student’s t-test, the Mann–Whitney test, or chi-square test with Yates correction. Pearson’s test was used to calculate correlation coefficients. Multiple stepwise regression analysis was performed to estimate the potential influence of various laboratory, biochemical factors and both SBP and MBP on LAVI and ASI. The following independence parameters were entered into the model: hemoglobin, sodium, potassium, calcium, phosphorus, Ca x P score, creatinine, urea, total protein, albumin, hs-CRP, T-chol, LDL-chol, HDL-chol, TGC, cTnT, NT-proBNP, ADMA. Probability values of <0,05 were accepted as significant.

## Results

### Baseline comparison

Clinical characteristics and laboratory measurements of the study population and control group are listed in Table 1. The heart rate and SBD, MBP were significantly higher among HD patients compared to controls, whereas DBP did not differ between study groups and control group.

**Table 1:** Pressure, heart rate and biochemical measurements collected in studied patients

**Parameter**	**HD**	**Controls**	**p**
Mean heart rate[beats/min]	74,40(±10,81)	73,47(±4,87)	0,402
Systolic blood pressure[mmHg]	139,4(±22,1)	123,7(±8,15)	<0,001
Diastolic blood pressure[mmHg]	72,98(±12,02)	74,15(±9,09)	0,387
Mean blood pressure[mmHg]	95,07(±14,28)	90,60(±3,74)	<0,001
Hemoglobin[g/dl]	11,98(±1,46)	13,89(±0,81)	<0,001
Sodium[mmol/l]	138,5(±2,5)	138,4(±1,7)	0,583
Potassium[mmol/l]	5,698(±0,735)	4,301(±0,278)	<0,001
Calcium[mmol/l]	2,259(±0,190)	2,364(±0,041)	0,007
Phosphorus[mmol/l]	1,991(±0,541)	1,09(±0,06)	<0,001
Ca X P[mg^2^/dl^2^]	55,86(±14,64)	31,28(±2,65)	<0,001
Creatinine [μmol/l]	777,0(±223,6)	81,33(±9,79)	<0,001
Urea[mmol/l]	24,39(±7,22)	4,233(±0,897)	<0,001
Total protein[g/l]	68,3(±0,52)	72,01(±0,28)	<0,001
Albumin[g/l]	3,935(±0,356)	4,636(±0,204)	<0,001
Hs-CRP[mg/l]	6,248(±0,23)	0,45(±0,09)	<0,001
T-chol[mg/dl]	189,7(±43,75)	190,5(±22,32)	0,437
LDL-chol[mg/dl]	114,3(±30,61)	112,0(±20,43)	0,504
HDL-chol[mg/dl]	41,51(±16,19)	56,7(±11,24)	<0,001
TGC[mg/dl]	170,8(±74,89)	109,1(±38,9)	<0,001
cTnT[(μg/l]	0,05(±0,011)	<0.01	<0,001
NT-proBNP[fmol/ml]	187,7(±96,2)	22,71(±17,23)	<0,001
ADMA[μmol/l]	1,031(±0,193)	0,772(±0,253)	<0,001

Baseline echocardiographic characteristic of the studied patients and controls are shown in Table 2. All diameters derived from a two-dimensionally guided M-mode echocardiography were significantly greater either in HD patients when compared to control group. Similarly parameters concerning left ventricle systolic and diastolic functions were markedly differ between study group and control group. SV and SI were decreased whereas CO and CI did not differ between them and controls. LVM and its index were significantly higher in HD patients. Patients exhibited significantly greater aortic stiffness expressed by higher value of ASI than in control group. Parameters concerning LA were also greater in HD patients than in control.

**Table 2:** Echocardiographic data

**Parameter**	**HD**	**Controls**	**P**
**Diameters of the heart**			
LVEDd[cm]	5,14(±0,59)	4,62(±0,44)	<0,001
LVESd[cm]	3,23(±0,54)	2,91(±0,36)	<0,001
PWDd[cm]	1,24(±0,24)	0,91(±0,05)	<0,001
PWSd[cm]	1,60(±0,23)	1,29(±0,06)	<0,001
IVSDd[cm]	1,44(±0,24)	0,94(±0,06)	<0,001
IVSSd[cm]	1,67(±0,28)	1,18(±0,07)	<0,001
**Parameters of LV mass**			
LVM[g]	253,9(±92,53)	154,6(±32,6)	<0,001
LVMI[g/m^2^]	146,5(±45,18)	97,14(±26,35)	<0,001
LVH[%]	64,8	0	<0,001
**Parameters of heart stroke**			
SV[ml]	70,39(±15,34)	79,46(±13,03)	0,009
SI [ml/beat/m^2^]	42,08(±11,19)	45,93(±4,22)	0,001
CO[l/min]	5,49(±0,81)	5,73(±0,49)	0,106
CI[l/min/m^2^]	3,24(±0,53)	3,31(±0,32)	0,126
**Parameters of systolic function**			
EF[%]	58,91(±6,21)	65,52(±3,87)	<0,001
FS[%]	31,54(±5,83)	38,9(±4,302)	<0,001
mFS[%]	15,34(±3,26)	20,12(±2,87)	<0,001
mFS/ESS[n]	0,186(±0,056)	0,218(±0,043)	0,002
**Parameters of diastolic function**			
E/A[n]	0,979(±0,256)	1,371(±0,135)	<0,001
IVRT[ms]	109,1(±22,73)	76,53(±14,04)	<0,001
DT[ms]	229,9(±45,12)	179,3(±27,41)	0,001
S/D[n]	1,49(±0,268)	1,22(±0,181)	<0,001
Relaxation abnormalities[%]	43,66	1,75	<0,001
**Parameters of left atrium**			
LAS[cm^2^]	23,59(±5,1)	19,35(±3,44)	<0,001
LAC[cm]	19,5(±4,58)	16,32(±3,07)	<0,001
LAmax[cm]	4,3(±0,59)	3,53(±0,27)	<0,001
LAV[ml]	62,52(±21,3)	36,82(±14,76)	<0,001
LAVI[ml/m^2^]	36,29(±10,92)	20,64(±6,77)	<0,001
**Parameters of aorta**			
ASI[n]	5,51(±1,32)	3,07(±1,09)	<0,001

**Figure 3:**
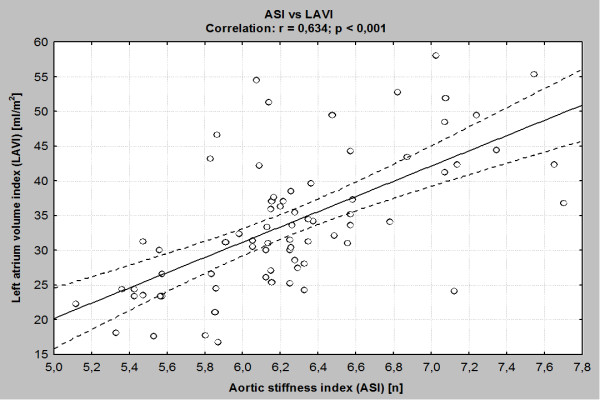
The association between ASI and LAVI.

The current study showed a significant correlation between the ASI and LAVI (Figure 3). We have also indicated a close, significant, relationship between the ASI and the indices of left ventricular systolic function as mFS/ESS, ESS and left ventricular diastolic function as E/A ratio, IVRT and S/D ratio. ASI also positively correlated with indicators of left ventricular hypertrophy as follows: LVM and LVMI (Table 3).

**Table 3:** Correlations between ASI [n] and echocardiographic parameters

**Parameter**	**R**	**P**
**LAV [ml]**	0,579	p < 0,001
**LAVI [ml/m**^**2**^**]**	0,634	p < 0,001
**ESV [ml]**	0,336	p = 0,024
**EDV [ml]**	0,372	p = 0,008
**LVM [g]**	0,532	P = 0,001
**LVMI [g/m**^**2**^**]**	0,543	p < 0,001
**E/A [n]**	−0,354	p = 0,009
**IVRT [msec]**	0,353	p = 0,021
**S/D [n]**	−0,402	p = 0,004
**ESS [%]**	0,442	p = 0,002
**mFS/ESS [n]**	−0,532	p < 0,001

### Cut-off values of selected echocardiographic indices

After the division into subgroups using mean LAVI value of 36,29 ml/m^2^, calculated for whole HD group, us cut-off value, in subgroups of LAVI < cut-off value were 38 patients (LAVI = 33,81 ± 7,93 ml/m^2^) and in subgroup of LAVI > cut-off value were 35 persons (LAVI 38,83 ± 9,37 ml/m^2^). Similarly study group was divided into two subgroups due to mean calculated value ASI of 5,61 as a cut-off value. The first of these subgroups of ASI < cut-off value consisted of 37 patients with ASI 4,15 ± 1,74 and in second subgroup of ASI > cut-off value were 36 subjects with ASI 7,19 ± 1,62. The comparison of subgroups created by cut-off values of LAVI and ASI with regarding to selected hemodynamic and laboratory data are shown in Table 4.

**Table 4:** The comparison of calculated cut-off value of ASI and LAVI to laboratory parameters

**Parameter**	**ASI > cut-off value n = 36**	**ASI < cut-off value n = 37**	**P**
ADMA[μmol/l]	1,134(±0,201)	0,881(±0,169)	0,002
cTnT[μg/l]	0,068(±0,016)	0,043(±0,09)	0,001
CRP[g/l]	6,92(±0,51)	5,57(±0,21)	0,007
Total chol(mg/dl)	229,4(±46,9)	162,4(±40,2)	0,01
LDL-chol(mg/dl)	128,2(±33,5)	101,1(±27,3)	0,02
NT-proBNP[fmol/ml]	195,8(±101,5)	172,9(±92,8)	0,03
Creatinine [μmol/l]	792,0(±229,9)	762,4(±218,8)	NS
Ca X P[mg^2^/dl^2^]	56,34(±15,14)	54,41(±13,81)	NS
	**LAVI > cut-off value n = 35**	**LAVI < cut-off value n = 38**	**P**
ADMA[μmol/l]	1,077(±0,197)	0,946(±0,159)	0,047
cTnT[μg/l]	0,063(±0,014)	0,048(±0,012)	0,001
CRP[mg/l]	6,520(±0,54)	5,905(±0,29)	0,03
Total chol[mg/dl]	201,4(±46,5)	181,2(±39,3)	NS
LDL-chol[mg/dl]	119,8(±31,3)	110,1(±26,8)	NS
NT-proBNP[fmol/ml]	231,2(±115,7)	136,9(±91,6)	0,001
Creatinine [μmol/l]	789,1(±231,4)	767,2(±207,4)	NS
Ca X P[mg^2^/dl^2^]	57,15(±16,08)	53,11(±12,55)	NS

### Multiple stepwise regression analysis

The ASI had been found independently and markedly associated with ADMA, cTnT, hs-CRP, T-chol, LDL-chol, but not with SBP and MBP in HD patients. Multiple stepwise regression analysis had been showed that the LAVI was independently associated with NT-proBNP and cTnT (Table 5).

**Table 5:** Factors influencing ASI and LAVI estimated by multivariate stepwise regression analysis

	**HD group**				
**dependent variable**	**independent variables**	**B**	**standard error**	**β**	**P**
ASI	ADMA	50,3	16,6	0,409	0,006
	cTnT	121,5	34,7	0,443	< 0,001
	CRP	4,92	1,83	0,281	0,002
	Total chol	0,805	0,237	0,340	0,003
	LDL-chol	1,427	0,428	0,329	0,004
LAVI	NT-proBNP	1,16	0,453	0,328	0,007
	cTnT	101,3	33,5	0,474	0,03

#### Reproducibility

Intraclass correlation for intraobserver variability was good for ASI measurements: 0,88; 0,77–0,95; 95% confidence interval. Intraclass correlations for intraobserver variability was also good for LAVI measurements: 0,85; 0,76–0,97; 95% confidence interval.

## Discussion

### LAVI and laboratory and echocardiographic parameters

Important observation of this study is that HD patients had greater LA dimension and therefore LAV and LAVI than control patients. These findings are important because it is well known that LAVI is an independent predictor of prognosis in HD patients providing information to traditional clinical and Doppler echocardiographic data [16]. We have adjusted LAV not by height, but by surface area. The adjustment to body size using height failed to nullify the gender influence on atrial size, unlike the adjustment to body surface area [17]. The diastolic dysfunction and increased LAV and LAVI in patients from hemodialysis group may in part be attributed to an increased LVMI as well as deteriorated elastic properties of the aorta.

In recent years, echocardiographic assessment of the LA and its relationship with cardiovascular risk have been revalued. LA size, particularly LAV has been recognized as a marker of diastolic dysfunction [18]. Current guidelines in the general population jointly issued by the American College of Cardiology, the American Heart Association, and the European Society of Cardiology consider the measurement of the LAV and LAVI as clinically relevant information [19]. Increased LAVI is a powerful predictor of mortality after acute myocardial infarction and provides prognostic information incremental to clinical data and conventional measure of left ventricle systolic and diastolic function [20]. LAVI is also useful index for prediction of adverse cardiovascular events and may predict first ischemic stroke and subsequent mortality [20]. Monitoring of left atrium size by echocardiography is also useful for predicting cardiovascular risk in patients with end-stage renal disease. Chan et al. [21] demonstrated that each mm/m^2^ increase in indexed LA diameter was associated with a small but significant risk of increased cardiovascular mortality [21]. Changes in LAV can also predict incident cardiovascular events in dialysis patients independent of the corresponding baseline measurement and of left ventricular mass [3].

In the present study NT-proBNP plasma level was significantly elevated in HD group. In literature there are conflicting data on the influence of glomerular filtration rate on BNP and NT-proBNP levels in ESRD. Tagore et al. [22] suggested that unlike NT-pro BNP, plasma BNP level is relatively independent of GFR. Most authors suggest to use NT-proBNP when patients with ESRD are evaluated. However data on NT-proBNP in renal dysfunction are more concordant but were derived from populations that included patients with impaired left ventricle function or heart failure [23]. This explain why is so difficult to account fully for the effect of coexistent cardiac disease, left ventricle dysfunction and volume overload, all conditions usually observed among patients with ESRD.

Our study has revealed for the first time highly significant correlation between LAVI and NT-proBNP among HD patients. Association between these two parameters was already described, but in other clinical settings. Kim et al. [24] documented the correlation of LAVI and level of NT-proBNP in patients with heart failure and a preserved ejection fraction. The combination of both LAVI and NT-proBNP also is more useful to stratify risk of sudden cardiac death than other clinical echocardiographic or biochemical variable [25]. The combination of these two parameters should be considered for predicting sudden cardiac deaths in patients with ESRD. Further studies are needed to assess the possible clinical importance of association between LAVI and NT-proBNP in such specific group of patients.

The higher values of LAVI with coexisting elevated plasma concentration of NT-proBNP may be a manifestation of so called cardio-renal syndrome [26]. In this clinical situation LA enlargement and higher NT-proBNP levels arising from heart disease, and greater preload which is partially caused by loss of elastic properties of aorta.

### ASI and laboratory and echocardiographic parameters

We evaluated that ASI was significantly higher in patients with ESRD than in subjects without renal dysfunction. It is well known that aortic elastic properties are affected by the risk factors for atherosclerosis. The coexistence of atherosclerosis and ESRD suggests there is the link between these two diseases [27]. Atherosclerosis is regarded as a combination of two separate diseases: atherosis and sclerosis. The aortic stiffness reflects sclerotic component and means mechanical properties of aortic wall. Calcium overload, which is characteristic feature for ESRD patients, and also observed in our patients especially before hemodialysis, is associated with aortic stiffening. Vascular calcification is detected either in the tunica intima or in the tunica media. Calcification in the intima is characteristic of most stages of atherosclerosis. Medial calcification is particularly common in patients with ESRD and may occur independently of atherosclerosis. Medial wall calcification increases vascular stiffness and reduces arterial compliance.

Multiple stepwise regression analysis has been shown that the ASI is independently associated with ADMA plasma concentration, troponin concentration, T-chol level, LDL-chol concentration and hs-CRP in HD patients. Our data demonstrate that HD patients have higher levels of ADMA. The ADMA is an endogenous competitive inhibitor of NO synthase. The ADMA competes with L-arginine. Therefore, NO levels decrease with increased ADMA levels. Recently, ADMA has been demonstrated to be a new and potentially independent marker of atherosclerosis and related cardiovascular disorders [28]. Endothelial dysfunction occurs when NO activity in the vascular tissue is decreased, resulting in decreased endothelium-dependent vasodilatation. ADMA is assumed to be a key regulator of NO synthase activity, notably of the endothelial NO synthase isoform. Thus, decreased NO activity might result in vasoconstriction and in that way increase aortic stiffness. To the best of our knowledge there are no data in the literature on ADMA concentration influence on aortic stiffness in patients with ESRD.

Accelerated vascular damage and defective vascular repair have been proposed as a mechanism for premature atherosclerosis and aortic stiffness, common findings among HD patients. Impairment of NO biosynthesis (e.g., by ADMA) or NO bioactivity (as with oxygen-derived free radicals) causes endothelial vasodilatation dysfunction. Thereby, the correlations between ASI and oxygen-derived free radicals promoters such as T-chol, LDL-chol, hs-CRP, observed in the present study, were no surprised. The deleterious effects of T-chol, LDL-chol and hs-CRP in part a consequence of decreased availability of endothelium cells-derived NO, include smooth muscle proliferation, collagen synthesis, and deterioration of elastin which may impair arterial compliance [29]. Taken together, our findings suggest at least 2 pathways that could lead to increase of aortic stiffness in HD subjects: wall calcification and endothelium dysfunction.

The significant correlation was also detected between ASI and several parameters characterizing left ventricle function. This include correlation between ASI and parameters characterizing systolic function as ESS and mFS/ESS and also between ASI and diastolic parameters: E/A ratio, IVRT and S/D ratio. We can only speculate about the mechanism linking aortic stiffness with left ventricle dysfunction. Loss of aortic wall compliance causes pressure elevation and abnormal pressure pattern that increases the afterload of the left ventricle. The result is development of left ventricle hypertrophy also strongly associated with aortic stiffness in studied groups.

### ASI and LAVI

The most important finding of echocardiographic aspect of the study was the significant correlation between ASI and LAVI in patients with ESRD. In our study, we found for the first time that HD patients with higher aortic stiffness have greater LAVI. There are several explanations for such association. Firstly, hypertension which is a common among patients with renal failure. It is well known that aortic stiffness is associated with LA size in hypertensive patients [30]. Additionally other cardiovascular risk factors present in large number in HD patients also can influence aortic stiffness and it that way stimulate LA enlargement [31]. All of them can modify unfavorably of aortic intima-media structure and provoke loss of elastic properties. In the other hand these factors increase shear stress and stimulate endothelium dysfunction which was also proved by us by demonstrating elevated ADMA plasma level. In result aortic compliance became markedly deteriorated.

It is well known that increase of aortic stiffness predict the degree of left ventricular diastolic dysfunction [32]. The main feature of this clinical state is elevation of left ventricle diastolic pressure. Both greater values of LAVI and high NT-proBNP plasma concentration are markers of elevated enddiastolic pressure as well as pressure measured in LA [33,34].

## Conclusions

The results of this study suggest, that aortic elastic properties are impaired in hemodialyzed patients. The levels of ADMA, T-chol, LDL-chol and hs-CRP may be a biomarkers or mediators of aortic stiffness in this clinical setting. Both LAVI and NT-proBNP concentration, signs of elevated left atrium pressure are markedly increased in hemodialyzed patients. The strong correlation between aortic stiffness index and LAVI, improved by association of specific biochemical markers with these echocardiographic indices, suggests, that there is the link between elastic properties of aorta and left atrium pressure in hemodialysed patients mediated by endothelial dysfunction.

### Limitations of the study

The limitations of this study include its cross-sectional nature design with relatively small number of subjects. We do not investigate whether ASI still predict LAVI after adjustments for other significant co-variables including biochemical factors like NT-pro BNB, ADMA, cTnT. Finally, aortic stiffness may be influenced by clinical features that were not controlled in this study, eg. hypertension, diabetes, smoking etc. It remains to be determined whether these variables may alter presented association between aortic stiffness and LAVI in hemodialysed patients.

## Abbreviations

HD: Hemodialysed patients; ESRD: End stage renal disease; IVSSd: Interventricular endsystolic septum diameter; IVSDd: Interventricular septum enddiastolic diameter; PWSd: Posterior wall systolic diameter; PWDd: Posterior wall diastolic diameter; LVEDd: Left ventricle enddiastolic diameter; LVESd: Left ventricle endsystolic diameter; LAmax: Left atrium maximal diameter; Aomax: Aortic maximal diameter; Aomin: Aortic minimal diameter; 4-CH: Four chamber; LAshort: Left atrium short diameter; LAlong: Left atrium longitudinal diameter; LAS: Left atrium surface; LAC: Left atrium circumference; SV: Left ventricle stroke volume; SI: Stroke index; CO: Cardiac output; CI: Cardiac index; EF: Ejection fraction; FS: Fractional shortening; LVM: Left ventricle mass; LVMI: Left ventricle mass index; ESS: Endsystolic stress; mFS: Midwall fractional shortening; LAV: Left atrium volume; LAVI: Left atrium volume index; ASI: Aortic stiffness index; E: Maximal velocity of early diastolic transmitral flow; A: Maximal velocity of late diastolic transmitral flow; IVRT: Isovolumetric relaxation time; S: Maximal systolic velocity in pulmonary veins; D: Maximal diastolic velocity in pulmonary veins; SBP: Systolic blood pressure; DBP: Diastolic blood pressure; MBP: Mean blood pressure; GFR: Glomerular filtration rate; hs-CRP: High sensivity C-reactive protein; T-chol: Total cholesterol; HDL-chol: HDL-cholesterol; TGC: Triglycerides; LDL-chol: LDL-cholesterol; MDRD: Modification of diet in renal disease (MDRD); cTnT: Cardiac troponin T; NT-proBNP: N-terminal fragment of brain natriuretic peptide; ADMA: Asymmetric dimethylarginine.

## Competing interests

The authors declare that they have no competing interests.

## Author’s contributions

TZ – carried out echocardiography, conceived of the study, and participated in its design, and coordination, and wrote the manuscript. AW - participated in its design, and coordination, and approved the manuscript. AK - conceived of the study, and participated in its design and coordination. AJ - analyzed and interpreted data conceived of the study, and participated in its design and coordination. All authors read and approved the final manuscript.

## Pre-publication history

The pre-publication history for this paper can be accessed here:

http://www.biomedcentral.com/1471-2261/12/100/prepub
